# Behavioural Determinants of Dietary Self‐Management in Chronic Kidney Disease: A Theory‐Informed Analysis to Inform Dietetic Interventions

**DOI:** 10.1111/jhn.70295

**Published:** 2026-07-02

**Authors:** Andrew Morris, Deborah Lycett, Riya Patel

**Affiliations:** ^1^ Health and Community Wellbeing Research Centre Coventry University Coventry UK; ^2^ Dietetic Department, Worcestershire Acute Hospitals NHS Trust Charles Hastings Way Worcester UK; ^3^ Renal Delivery Network, NIHR West Midlands Regional Research Delivery Network, The Royal Wolverhampton Trust New Cross Hospital, Wednesfield Wolverhampton UK; ^4^ Diabetes Research Department, NIHR East Midlands Applied Research Centre Leicester General Hospital Leicester UK

**Keywords:** APEASE, behaviour change wheel, nutrition, renal, theoretical domains framework

## Abstract

**Objective:**

To identify the key behavioural determinants that influence dietary self‐management among adults with CKD, using the Theoretical Domains Framework and Behaviour Change Wheel to guide development of practical dietary interventions for clinical practice and research trials.

**Methods:**

A theory‐informed secondary analysis of a published qualitative meta‐synthesis that included 92 studies and 2924 adults with CKD from 23 countries. Themes from the meta‐synthesis were mapped to Theoretical Domains Framework (v2) and linked to Behaviour Change Wheel intervention functions and behaviour change techniques. Potential behaviour change intervention strategies were then evaluated using the APEASE criteria (Affordability, Practicability, Effectiveness/Cost‐effectiveness, Acceptability, Side‐effects/Safety, Equity).

**Results:**

Twelve of the 14 Theoretical Domains Framework (v2) domains were identified, indicating that dietary self‐management in CKD is influenced by a broad range of cognitive, emotional, social, and environmental factors. Key barriers included inconsistent or conflicting dietary advice (Knowledge; Environmental Context and Resources), limited practical guidance (Skills; Behavioural Regulation), and emotional distress and social disruption associated with dietary change (Emotion; Social Influences). Trust in healthcare professionals and experiential learning also shaped adherence (Beliefs about Capabilities and Consequences). Optimism and Intentions domains were not evident. Mapping to the Behaviour Change Wheel identified Education, Enablement, Environmental Restructuring, Training, Persuasion, Incentivisation, and Modelling as relevant intervention functions. APEASE assessment suggested that Education and Enablement were the most feasible, acceptable, and scalable approaches for clinical implementation, while more complex components requiring additional resources or coordination (e.g., training and system‐level changes) were comparatively less practical.

**Conclusions:**

Dietary self‐management in CKD is influenced by multiple behavioural determinants beyond knowledge alone. Interventions should therefore integrate education with skills training, confidence building, social support, and environmental adaptation. Embedding these multi‐component strategies within clinical nutrition practice and research may enhance self‐management and improve patient engagement across the CKD care pathway.

## Summary

- Dietary self‐management in chronic kidney disease is shaped by far more than knowledge alone. Emotional distress, social disruption, conflicting advice, and limited practical skills all act as significant barriers, and effective dietetic care needs to address these alongside nutritional information.
- Education combined with enablement (practical skills training, confidence building, and tailored problem‐solving) emerged as the most feasible and acceptable approach for routine clinical practice and should form the core of dietary interventions for adults with CKD.
- Consistent, coordinated dietary messaging from the multidisciplinary team, paired with social support and adaptation of the patient's home and food environment, is likely to improve adherence and engagement more than information‐giving alone.

## 1 Introduction

Dietary management is an important part of chronic kidney disease (CKD) care, with interventions for controlling potassium, phosphate, sodium, and fluid intake aimed at mitigating symptoms, helping slow disease progression, and reducing complications [1, 2, 3, 4, 5, 6, 7]. However, these dietary recommendations are multifaceted, often changing over time with kidney function, and conflict with other dietary guidance for comorbid conditions such as diabetes and cardiovascular disease [8]. As a result, people living with CKD frequently report difficulties understanding, implementing, and sustaining dietary recommendations, which may negatively impact their nutritional status [9], quality of life [8], and clinical outcomes [10].

Systematic reviews examining how best to support people with CKD in self‐managing their dietary recommendations reported behaviour change intervention effectiveness remain unclear, primarily due to inadequacies in design. For example, Peng et al. (2019) reported that intervention efficacy remains uncertain given randomised controlled trials lacked behavioural change frameworks to underpin dietary self‐management interventions [11]. Although Brown et al. (2021) found that a limited number of controlled trials showed benefits to diet quality and renal risk factors, the evidence reviewed was inconsistent in its design and reporting of behaviour change theory [12].

From a renal dietetic perspective, it has been reported certain behaviour change theories have been shown to be effective in nutritional interventions, such as the Trans‐theoretical Model and Social Cognitive Theory [13]. However, even when interventions are informed by established theoretical models, such as Social Cognitive Theory or the Transtheoretical Model, these individual frameworks may not encompass all relevant factors influencing dietary behaviours in CKD [14, 15]. For instance, Social Cognitive Theory emphasises self‐efficacy and observational learning but may overlook environmental constraints like food availability or healthcare system barriers. Similarly, the Transtheoretical Model focuses on stages of behavioural readiness yet may fail to address social, cultural, or physiological factors unique to CKD patients. Consequently, relying on a single or narrow theoretical lens can exclude critical determinants of behaviour, limiting the overall effectiveness of healthcare interventions [16].

Context plays a critical role in effective dietary self‐management, and integrating contextual considerations into clinical practice may substantially enhance dietary adherence. Qualitative studies indicate that clinicians often overlook patients' realities, resulting in advice that is inconsistent, overly technical, or culturally misaligned factors that foster confusion, erode trust, and contribute to disengagement from dietary guidance [8]. Moreover, adherence is influenced not only by nutritional knowledge but also by emotional responses, social dynamics, environmental constraints, and individuals' beliefs regarding their capability and the anticipated consequences of dietary change. These contextual determinants are seldom incorporated into traditional nutrition‐counselling approaches, limiting their effectiveness [8].

A recent systematic review and co‐created qualitative synthesis by Morris et al. (2025) [8] sought to address this gap by exploring the experiences of adults with CKD in managing complex dietary advice, with the aim of generating patient‐informed themes to guide future intervention development. This review synthesised qualitative evidence from 92 studies to identify key barriers and facilitators to nutritional self‐management, highlighting the multifactorial and context‐dependent nature of dietary behaviours in CKD. However, given the scale and complexity of the synthesis, theory‐based mapping to behaviour change frameworks was not undertaken within the primary review and was instead reserved as a subsequent, pre‐specified analytical phase. As a result, while the review provided in‐depth, patient‐centred insights, the findings were not explicitly translated into a comprehensive behaviour change framework, limiting their direct applicability to intervention design.

Therefore, a secondary analysis of this qualitative synthesis is warranted to translate these experiential insights into theory‐informed intervention strategies. In this context, the present study mapped the findings of the Morris et al. (2025) [8] meta‐synthesis on to the Theoretical Domains Framework (v2) [17] and the Behaviour Change Wheel [18]. By mapping, we aimed to identify theory‐based targets for intervention development that reflect the real‐world needs and preferences of people who need to self‐manage CKD through diet.

Through applying the APEASE criteria (Affordability, Practicability, Effectiveness/Cost‐effectiveness, Acceptability, Side‐effects/Safety, and Equity), we assessed the acceptability, feasibility, equity, and likely effectiveness of these interventions to inform recommendations for practice and research in supporting dietary self‐management.

## 2 Methods

The original systematic review aimed to synthesise qualitative evidence on the experiences of adults with CKD in managing dietary recommendations, with a focus on identifying barriers, facilitators, and implications for intervention development. The review generated inductive descriptive and analytical themes that captured the complexity of dietary self‐management in CKD.

As specified as a priori in the PROSPERO‐registered protocol (CRD 42023440615), the synthesis was designed to extend beyond thematic interpretation by incorporating a theory‐based analysis. Specifically, the protocol outlined that the final analytical themes would be mapped to the Theoretical Domains Framework (v2) [17], with subsequent identification of intervention functions using the Behaviour Change Wheel [18].

The present study represents this planned analytical phase, undertaken as a secondary analysis of the completed qualitative synthesis. While the primary review focused on developing an in‐depth understanding of patient experiences, this secondary analysis applies a behavioural science lens to systematically translate those findings into theoretically informed determinants of behaviour and potential intervention strategies. This approach enables a more explicit link between qualitative evidence and intervention design, addressing a recognised gap in the application of behavioural theory within CKD dietary self‐management research [12].

Conducting this analysis as a distinct phase allowed for a more rigorous and transparent application of the Theoretical Domains Framework (v2) [17] and Behaviour Change Wheel [18] frameworks, ensuring that the mapping process was systematic, theory‐driven, and grounded in the fully developed analytical themes derived from the original meta‐synthesis [8].

Ethical approval was not sought as this was a secondary analysis of published qualitative data.

### 2.1 Design

A theory‐informed secondary analysis of qualitative data using the Theoretical Domains Framework (v2) as detailed by Cane et al. [19] was undertaken. The epistemological rationale for using deductive theory mapping on secondary qualitative syntheses was that behaviour determinants were not explicitly analysed in Morris et al. (2025) [8].

A critical‐realist pragmatism epistemology was deemed suitable as we treated the qualitative synthesis as a credible representation of patients' experiences, while acknowledging that behavioural theories can help identify and structure the psychological, emotional, social, and environmental determinants underlying those experiences. By taking this approach, our aim was to extend our understanding of behaviour mechanisms.

We mapped onto the Theoretical Domains Framework (v2) [17] to identify behavioural determinants relevant to dietary self‐management in adults with CKD. We then mapped these determinants onto the Behaviour Change Wheel [18]. This approach allowed for a structured interpretation of patients lived experiences to inform evidence‐based intervention development underpinned by appropriate evidence‐based behaviour change theory taxonomy [19].

While the Capability, Opportunity, Motivation – Behaviour model [18] provides a high‐level behavioural system, the Theoretical Domains Framework (v2) [17] offers greater conceptual specificity across behavioural determinants. As Theoretical Domains Framework (v2) [17] domains are theoretically mapped onto the Capability, Opportunity, Motivation – Behaviour model, conducting an additional explicit Capability, Opportunity, Motivation – Behaviour model mapping step was not considered necessary. Instead, the use of Theoretical Domains Framework (v2) [17] enabled a more detailed and nuanced analysis, while remaining fully aligned with the Behaviour Change Wheel framework.

### 2.2 Theoretical Domains Framework and Behaviour Change Wheel

The Theoretical Domains Framework (v2) [17] is a validated integrative framework that consolidates constructs from multiple behaviour change theories into 14 domains relevant to implementation and intervention design [20, 21] (Supporting Information Table S1). These domains include knowledge, skills, social influences, beliefs about capabilities and consequences, emotion, environmental context, and behavioural regulation. The Theoretical Domains Framework has been widely used to explore behavioural determinants in health care settings, including in the context of patient self‐management [19, 22, 23].

**Figure 1:**
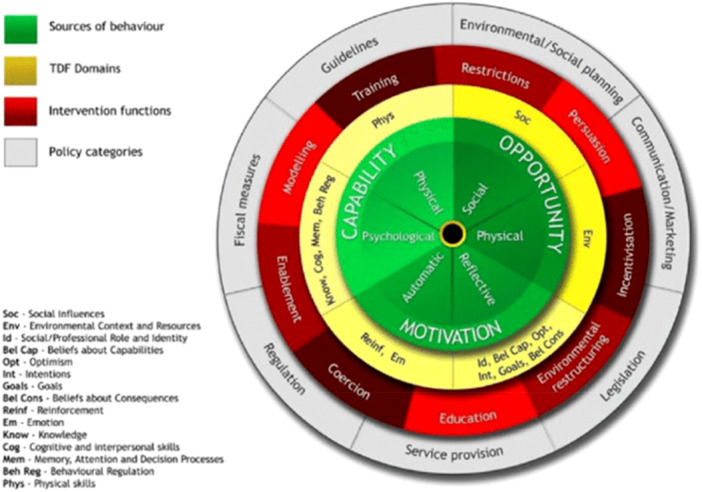
The Behaviour Change Wheel [18] (Figure 1 reproduced without change).

The Behaviour Change Wheel [18] offers a comprehensive framework for designing and evaluating behaviour change interventions and contains nine intervention functions such as education, persuasion, and environmental restructuring that can be employed to influence behaviour (Figure 1). The Behaviour Change Wheel [18] synthesises 19 existing behaviour change frameworks into a coherent model, facilitating systematic identification of intervention targets and appropriate strategies. Its integration with complementary tools, including the Theoretical Domains Framework (v2) and the Behaviour Change Technique Taxonomy (BCTTv1), supports both the development and reporting of interventions. This theoretically grounded approach has been increasingly applied across diverse health contexts, including chronic disease management and dietary behaviour modification, providing a systematic framework to improve the design, implementation, and reproducibility of behaviour change interventions [24, 25].

### 2.3 Assessing Behaviour Change Wheel Interventions Against APEASE

The APEASE criteria (Affordability, Practicability, Effectiveness/Cost‐effectiveness, Acceptability, Side‐effects/Safety, and Equity) was applied to each Behaviour Change Wheel [18] intervention function identified for the dietary self‐management of chronic kidney disease. This assessment was based on the mapped Theoretical Domains Framework domains (v2) [17], Behaviour Change Wheel intervention functions [18], and relevant behaviour change techniques.

Each intervention was systematically reviewed against the six APEASE dimensions and coded to indicate the strength of alignment. This process provided a transparent, evidence‐informed rationale for selecting interventions that are feasible, acceptable, cost‐effective, and equitable for people living with CKD who self‐manage dietary recommendations.

### 2.4 Data Source

We used a published qualitative meta‐synthesis that included 92 studies exploring the experiences of 2924 adults with CKD, from 23 countries, in self‐managing dietary advice [8]. No new primary data was analysed. The systematic review's search strategy and the quality appraisal of the 92 included studies have been reported elsewhere [8].

The original synthesis used thematic analysis to generate interpretive themes regarding how patients interact with, adapt to, and make sense of dietary recommendations and their nutritional self‐management experiences. The systematic review took a collaborative approach with patients, healthcare professionals and commissioners to make the results of the synthesis pragmatic and relevant to clinical practice. Three themes and eight sub‐themes examined issues such as the timing and quality of dietary advice, the emotional burden of self‐management, social and cultural influences, and the negotiation of dietary changes in daily life.

### 2.5 Mapping Process

One researcher (AM) reviewed the meta‐synthesis findings and coded the interpretive themes against the 14 Theoretical Domains Framework (v2) domains [17]. Mapping was conducted deductively, guided by Theoretical Domains Framework (v2) [17] definitions and examples outlined by Cane et al. [19] and Michie et al. [18]. Where relevant, themes were mapped to multiple domains to capture overlapping constructs. A second researcher (RP) reviewed all code – domain mappings. Any discrepancies were resolved through discussion and consensus, and when consensus could not be reached a third researcher was invited to resolve conflicts (DL). Domains were then linked to potential intervention functions using the Behaviour Change Wheel [18] and assessed against the APEASE criteria (Affordability, Practicability, Effectiveness/Cost‐effectiveness, Acceptability, Side‐effects/safety, Equity).

## 3 Results

### 3.1 Mapping the Meta‐Synthesis to the Theoretical Domains Framework

Mapping of the meta‐synthesis themes onto the Theoretical Domains Framework (v2) [17] identified a comprehensive range of behavioural determinants influencing dietary self‐management in individuals with CKD. The Theoretical Domains Framework (v2) [17] comprises 14 domains encompassing psychological, emotional, social, and environmental factors that can influence behaviour, including Knowledge, Skills, Social/Professional Role and Identity, Beliefs about Capabilities, Beliefs about Consequences, Reinforcement, Emotion, and Environmental Context and Resources. The interpretive themes from the original meta‐synthesis, Navigating Dietary Advice (subthemes: Receiving Advice, Quality of Advice, Tailoring of Advice), Adjusting to a New Life (subthemes: Mourning an Old Life, Trial and Error, Enough is Enough [Re]gaining Control), and Diet as a Social Construct (subthemes: Social Influences on Management, Stigma and Isolation) were each mapped to multiple Theoretical Domains Framework (v2) [17] domains with their rationale (Table 1). Across all themes, 12 of the 14 Theoretical Domains Framework (v2) [17] domains were represented, indicating a multifaceted interplay of psychological, social, and environmental determinants. The domains Optimism and Intentions were not represented.

**Table 1:** Meta‐synthesis mapped to the Theoretical Domains Framework (v2).

Theme 1 – Navigating dietary advice		
Receiving advice		
Area identified	Mapped TDF domains	Justification
Late/repetitive advice; preference for early guidance	Environmental context and resources; memory, attention and decision processes	Timing and delivery of information influence both accessibility of care and cognitive processing of dietary advice.
Seeking information; navigating conflicting advice	Knowledge; social influences	Patients actively seek information from multiple sources and must reconcile inconsistencies across social and professional inputs.
Empathetic vs paternalistic communication	Social influences	Interpersonal communication style shapes trust, engagement, and receptivity to dietary advice.
Valuing peer support	Social influences	Peer interactions provide both informational and emotional support, influencing dietary behaviours.
Preference for convenient, low‐cost modalities (e.g., calls, texts)	Environmental context and resources	Accessibility and mode of delivery affect engagement with dietary support.
Preference for blood tests as feedback/motivation	Reinforcement	Objective clinical feedback reinforces behaviour through perceived consequences.

Quality of advice		
Area identified	Mapped TDF domains	Justification
Complexity of advice (changing advice), variable confidence	Knowledge; beliefs about capabilities	Understanding of dietary advice influences confidence in the ability to implement recommendations.
Conflicting advice from HCPs and online	Knowledge; social influences	Inconsistencies across sources reduce clarity and influence behaviour through social credibility.
Desire for food‐based over nutrient‐based guidance	Skills	Preference for practical, actionable guidance reflects the need for applied dietary skills.
Challenges managing multiple long‐term conditions	Environmental context and resources	Competing health demands create structural barriers to dietary adherence.

Tailoring of advice		
Area identified	Mapped TDF domains	Justification
Need for personalised advice based on lifestyle and kidney function	Environmental context and resources; beliefs about capabilities	Tailored advice improves feasibility and enhances confidence in implementation.
Generic advice from non‐dietitians	Social/professional role and identity	Professional roles influence the specificity and perceived credibility of dietary guidance.
Lack of integration across comorbidities	Environmental context and resources	Fragmented care limits the ability to implement coherent dietary strategies.
Cultural inappropriateness of dietary advice	Environmental context and resources	Cultural mismatch reduces relevance and acceptability of dietary recommendations.

Theme 2 – Adjusting to a new life		
Mourning an old life		
Area identified	Mapped TDF domains	Justification
Emotional grief and social withdrawal	Emotion; social influences	Emotional responses to illness impact social engagement and behaviour.
Dietary self‐management as life‐prolonging vs health‐improving	Beliefs about consequences	Framing of outcomes influences motivation and engagement.
Dialysis as emotionally distressing	Emotion	Emotional burden of treatment affects adherence.

Trial and error		
Area identified	Mapped TDF domains	Justification
Learning through food‐body response	Skills; behavioural regulation	Experiential learning supports skill development and behavioural adjustment.
Lack of symptoms undermining motivation	Beliefs about consequences	Absence of immediate feedback reduces perceived importance of adherence.
Empowerment through acceptance	Beliefs about capabilities	Acceptance enhances perceived control and self‐efficacy.
Deferring decisions to HCPs	Social/professional role and identity	Perceived role boundaries reduce autonomy in dietary decision‐making.

(Re)gaining control		
Area identified	Mapped TDF domains	Justification
Rebellious behaviours, testing limits	Goals, emotion; behavioural regulation	Emotional responses drive behavioural experimentation and boundary testing.
Justifying indulgences or partial adherence	Behavioural regulation	Individuals regulate behaviour through flexible adherence strategies.
Disbelief in advice necessity	Beliefs about consequences	Scepticism regarding outcomes undermines adherence.

Theme 3 – Diet as a social construct		
Social influences on management		
Area identified	Mapped TDF domains	Justification
Family as supporters or barriers	Social influences	Family dynamics directly shape dietary behaviours.
Social tensions in maintaining relationships	Social influences; emotion	Emotional strain within relationships affects adherence.

Stigma and isolation		
Area identified	Mapped TDF domains	Justification
Social isolation and avoidance	Social influences; emotion	Social exclusion impacts emotional wellbeing and dietary engagement.
Managing social dining situations (pretending to eat, eating to please others)	Social influences; behavioural regulation	Individuals adapt behaviour to balance social expectations and dietary needs.

Abbreviation: TDF, Theoretical Domains Framework (v2).

### 3.2 Navigating Dietary Advice

In the theme *Navigating Dietary Advic*e, patients frequently reported receiving dietary advice as delayed, repetitive, or inconsistently delivered. These experiences were mapped onto the Theoretical Domains Framework (v2) [17] domains of Environmental Context and Resources, Knowledge, and Memory, Attention, and Decision Processes. Patients often sought clarification from multiple sources, including family members, healthcare professionals, and online forums, underscoring the influence of Social Influences and the need for improved Cognitive and Interpersonal Skills. Communication style was critical, with empathetic, collaborative approaches perceived as empowering which mapped onto the domains of Beliefs about Capabilities and Emotion, whereas paternalistic interactions undermined trust. Peer support emerged as a valued source of information and reassurance, reinforcing the importance of Social Influences and Social/Professional Role and Identity. Patients also emphasised the practicality of advice delivery, including a preference for digital or low‐burden modalities and the motivational role of objective feedback (e.g., blood test results) which mapped onto the domains of Reinforcement and Beliefs about Consequences.

Participants commonly expressed difficulties navigating multifaceted, and sometimes contradictory, dietary guidance, especially when advice failed to reflect their comorbidities, lifestyle, or cultural background. These experiences mapped to the Beliefs about Capabilities, Knowledge, Environmental Context and Resources, and Behavioural Regulation domains. There was a strong preference for food‐based rather than nutrient‐based advice, with patients seeking actionable strategies that could be integrated into daily life. The perceived authority and dietary knowledge of the advice‐giver (e.g., dietitian vs. non‐specialist) influenced credibility and adherence reflecting the role of Social/Professional Role and Identity.

### 3.3 Adjusting to a New Life

The emotional burden of CKD and its dietary implications was consistently emphasised in the theme *Adjusting to a New Life*, with subthemes around grief, distress, isolation, and loss of social normality which mapped to Emotion, Social Influences, and Beliefs about Consequences domains. Dietary adherence was influenced by how patients interpreted the purpose of diet changes, whether this was to prolong life, improve wellbeing, or avoid complications. Dialysis was framed as a turning point that either motivated or overwhelmed patients emotionally which mapped to the Reinforcement domain.

Patients frequently reported dietary self‐management as an evolving process of 'trial and error,' wherein personal experimentation shaped their beliefs about what was feasible or necessary for their CKD. This subtheme was associated with Behavioural Regulation, Beliefs about Capabilities, and Cognitive and Interpersonal Skills. A lack of overt symptoms following dietary advice non‐compliance sometimes reduced motivation, highlighting the role of Reinforcement and Beliefs about Consequences. While some individuals deferred decision‐making to healthcare professionals, others reported reclaiming control through adaptive coping strategies, aligning with the Goals, Emotion, Social/Professional Role and Identity domains.

### 3.4 Diet as a Social Construct

In the theme *Diet as a Social Construct*, diet was experienced as a socially embedded practice, influenced by family dynamics, cultural norms, and social expectations. Participants reported difficulty navigating social occasions, concealing dietary needs, or feeling stigmatised by restrictions which reflected Social Influences, Emotion, Behavioural Regulation, and Beliefs about Consequences. The social impact of dietary adherence was a recurrent contextual barrier.

The Theoretical Domains Framework (v2) [17] analysis was mapped to the Behaviour Change Wheel intervention functions with example behaviour change techniques (BCTs) relevant to clinical practice [18] (Table 2). Key determinants included gaps in knowledge and practical skills, lack of multidisciplinary role clarity, and the influence of patient beliefs, emotions, and social networks. Environmental factors such as dietetic service accessibility, food availability, and cultural appropriateness further shaped dietary behaviours. The mapping indicated that effective interventions would require multi‐component approaches targeting capability, opportunity, and motivation, rather than education alone (Table 2). These interventions were mapped to the Behaviour Change Taxonomy [18, 19].

**Table 2:** Theoretical Domains Framework (v2) mapped to the Behaviour Change Wheel and Behaviour Change Taxonomy v1 to identify interventions.

TDF domain	Subthemes	BCW intervention function	Examples of BCT taxonomy v1	Intervention strategy (What to do and why)
Knowledge	Seeking information; navigating conflicting advice; conflicting advice from HCPs and online	Education, enablement	4.1 Instruction on how to perform behaviour 5.1 Information about health consequences; 9.1 Credible source	Provide clear, consistent dietary guidance delivered by a nutrition‐credible healthcare professional, including specific dietary instructions and explanations of health consequences. This reduces confusion and supports informed decision‐making by improving knowledge and trust in information sources.
Skills	Learning through food‐body response; desire for food‐based over nutrient‐based guidance	Training, enablement	8.1 Behavioural practice/rehearsal; 4.4 Behavioural experiments; 6.1 Demonstration of behaviour	Provide structured, food‐based learning using guided experiments (e.g., trying specific food swaps) combined with objective feedback (e.g., blood results, weight, fluid status) and self‐monitoring tools. Encourage patients to reflect on their food choices and symptoms. Where symptoms are absent, use proxy indicators and clinician feedback to make outcomes visible, helping patients build skills through observable cause–effect relationships rather than relying on physical symptoms.
Social/Professional role and identity	Generic advice from non‐dietitians; deferring decisions to HCPs	Modelling, environmental restructuring, enablement	6.2 Social comparison; 12.2 Restructuring the social environment; 3.1 Social support (unspecified)	It is recommended dietitians lead dietary guidance, supported by consistent messaging across the MDT, and provide opportunities to observe and discuss others' self‐management (e.g., peer review). This clarifies roles, reduces reliance on non‐specialist advice, and supports patient autonomy in decision‐making.
Beliefs about capabilities	Confidence varies with how comprehensible and actionable the advice is; empowerment through acceptance	Enablement, persuasion	1.4 Action planning; 1.2 Problem solving; 15.1 Verbal persuasion about capability	Support patients to develop personalised action plans, problem‐solve barriers, and provide reassurance about their ability to manage diet. This builds self‐efficacy by making advice understandable, achievable, and reinforcing confidence.
Beliefs about consequences	Lack of symptoms undermining motivation; dietary self‐management as life‐prolonging vs health‐improving; disbelief in advice necessity	Persuasion, education	5.2 Salience of consequences; 5.6 Information about emotional consequences; 9.1 Credible source	Provide clear, credible explanations of short‐ and long‐term consequences, including emotional and quality‐of‐life impacts, to make risks and benefits more tangible. This strengthens motivation where symptoms are not immediately apparent.
Reinforcement	Preference for blood tests as feedback/motivation	Incentivisation, enablement	2.2 Feedback on behaviour; 2.7 Feedback on outcome(s) of behaviour; 10.4 Social reward	Use regular feedback (e.g., blood test results) to show the link between diet and health outcomes, combined with positive reinforcement from the MDT. This strengthens behaviour through observable and meaningful outcomes.
Goals	Rebellious behaviours, testing limits	Enablement persuasion modelling	1.1 Goal setting (behaviour); 1.3 Goal setting (outcome); 1.5 Review behaviour goal(s); 1.7 Review outcome goal(s); 1.4 Action planning; 1.2 Problem solving; 6.1 Demonstration of behaviour; 9.1 Credible source	Collaboratively set realistic behavioural and outcome goals, with regular review and adjustment, and demonstrate achievable strategies. This channels resistance into structured goal setting and supports autonomy while maintaining adherence.
Memory, Attention and decision processes	Late/repetitive advice; preference for early guidance	Enablement	1.1 Goal setting (behaviour);1.3 Goal setting (outcome); 1.5 Review behaviour goal(s)	Deliver dietary advice early and reinforce it over time, supported by goal‐setting and regular review. This improves recall, supports decision‐making, and embeds behaviour through repetition.
Environmental context and resources	Preference for convenient, low‐cost modalities (e.g., calls, texts); need for personalised advice based on lifestyle and kidney function; cultural inappropriateness of dietary advice; challenges managing multiple LTCs; lack of integration across comorbidities	Environmental restructuring, education	7.1 Prompts/cues; 4.1 Instruction on how to perform behaviour	Provide accessible, tailored dietary support (e.g., phone/text follow‐ups), with culturally appropriate and personalised advice, and use prompts/reminders to support adherence. This increases feasibility by aligning interventions with real‐world constraints. Importantly it provides support when eating outside the home and navigating social situations around food.
Social influences	Empathetic vs paternalistic communication; valuing peer support; family as supporters or barriers; social tensions in maintaining relationships; managing social dining situations (pretending to eat, eating to please others) social isolation and avoidance	Enablement, environmental restructuring, modelling	5.1 Information about social and environmental consequences; 3.1 Social support (unspecified); 3.2 Social support (practical); 2.2 Restructuring the social environment; 13.2 Framing/reframing; 8.2 Behaviour substitution; 3.3 Social support (emotional)	Promote empathetic, patient‐centred communication, involve family and peers as support and provide strategies to manage social situations (e.g., food substitutions, reframing expectations). This leverages social context to support rather than hinder adherence.
Emotion	Emotional grief and social withdrawal; dialysis as emotionally distressing	Persuasion, enablement, Education	11.2 Reduce negative emotions; 3.3 Social support (emotional); 5.6 Information about emotional consequences	Provide emotional support and coping strategies, alongside information about emotional responses to illness, to help patients manage distress and remain engaged with dietary behaviours.
Behavioural regulation	Learning through food‐body response; justifying indulgences or partial adherence; managing social dining situations (pretending to eat, eating to please others)	Training, enablement, education, persuasion, environmental restructuring	2.3 Self‐monitoring of behaviour; 2.4 Self‐monitoring of outcomes of behaviour; 13.2 Framing/reframing; 8.2 Behaviour substitution;12.2 Restructuring the social environment	Support self‐monitoring of diet and outcomes (e.g., symptoms, blood test results), encourage adaptive reframing of lapses, and provide practical strategies for managing social eating. This strengthens ongoing self‐regulation and flexible adherence.

*Note:* Mapping of meta‐synthesis to the Theoretical Domains Framework (v2) (TDF), Behaviour Change Wheel (BCW) intervention functions, and example Behaviour Change Techniques (BCTs) relevant to dietary management of CKD. Themes were derived from a meta‐synthesis and coded into TDF domains, then linked to BCW functions and corresponding BCTs from the BCT Taxonomy v1. The 'Justification for Intervention' column summarises the rationale for selecting intervention functions and BCTs based on contextual factors identified in the mapping process.

The APEASE assessment for dietary interventions in the self‐management of CKD (Table 3) showed Education and Enablement interventions for improving knowledge are highly cost‐effective, easily delivered via digital or group formats, and well accepted by patients and multidisciplinary teams, with minimal safety concerns. Skills training is moderately resource‐intensive but practical when integrated into routine sessions and strongly supports self‐management behaviours. Interventions targeting social/professional roles and beliefs about capabilities rely on modelling, persuasion, or enablement, are low‐cost, and enhance patient empowerment and self‐efficacy, particularly when multidisciplinary teams are supportive of this approach. Reinforcement and goal‐setting strategies are effective for adherence and behaviour regulation, though equitable access may require alternatives to technology‐based delivery. Environmental and social interventions, including restructuring contexts and leveraging peer or community support, address structural and emotional barriers, are generally practical, and have high potential to reduce disparities if culturally tailored. Overall, the interventions reviewed demonstrate high acceptability, minimal risk, and meaningful opportunities to promote equity and sustain behaviour change.

**Table 3:** APEASE assessment for dietary interventions in the self‐management of CKD.

TDF domain	BCW intervention function(s)	Affordability	Practicability	Effectiveness/Cost‐effectiveness	Acceptability	Side‐effects/Safety	Equity
Knowledge	Education, enablement	High–low‐cost materials once developed	Easily delivered via digital, group, or 1:1	Strong evidence for improving knowledge and decision‐making	High among patients and MDT	Minimal risk	Improves equity if culturally adapted and consistent
Skills	Training, enablement	Moderate–requires trained staff/time	Feasible within existing education sessions	Highly effective for building self‐management skills, especially with feedback	High with interactive, food‐based approaches	Minimal	Reduces disparities if universally accessible
Social/Professional role and identity	Modelling, environmental restructuring, enablement	Low–moderate (training, MDT coordination)	Requires consistent MDT collaboration	Effective for improving role clarity and patient autonomy	High if MDT aligned	Low	Promotes equity through standardised messaging
Beliefs about capabilities	Enablement, persuasion	Low (planning and coaching)	Easily embedded into routine care	Strong evidence for improving self‐efficacy and adherence	High	Minimal	Particularly beneficial for lower‐confidence groups
Beliefs about consequences	Persuasion, education	Low (communication‐based)	Easily integrated into consultations	Effective when linked to tangible/proxy outcomes (e.g., blood test results, fluid status, dry weight)	High	Minimal	Needs cultural tailoring for relevance
Reinforcement	Incentivisation, enablement	Low–moderate (feedback systems)	Feasible via clinic review or simple tracking tools	Strong evidence for reinforcing behaviour via feedback loops	High if feedback is meaningful	Minimal	Requires low‐tech options to avoid widening inequalities
Goals	Enablement, persuasion, modelling	Low cost	Highly feasible with structured tools	Effective when goals are personalised and regularly reviewed	High	Minimal	Supports equity through individual tailoring
Memory, attention and decision processes	Enablement, environmental restructuring	Low (reminders, prompts)	Highly practical via digital or clinic systems	Effective for improving recall and sustaining behaviours	High	Minimal	Improves equity if accessible across formats
Environmental context and resources	Environmental restructuring, education	Variable (depends on resources)	Feasible using existing services/community resources	Effective for addressing structural and contextual barriers	High if personalised	Minimal	High potential to reduce health inequalities
Social influences	Enablement, environmental restructuring, modelling	Low–moderate (peer/family interventions)	Feasible with coordination	Effective for leveraging social support and norms	High	Minimal	Can reduce isolation and support underserved groups
Emotion	Persuasion, enablement, education	Low–moderate (support interventions)	Feasible within MDT or peer support	Effective for addressing emotional barriers to adherence	High	Minimal	Particularly beneficial for vulnerable groups
Behavioural regulation	Training, enablement, environmental restructuring	Low–moderate (monitoring tools/support)	Feasible via apps, diaries, or clinic review	Strong evidence for improving adherence through self‐monitoring and adaptation	High	Minimal	Requires low‐tech alternatives for equitable access

Abbreviations: BCW, Behaviour Change Wheel; TDF, Theoretical Domains Framework (v2).

## 4 Discussion

Our theory‐informed secondary analysis offers novel insights into the behavioural determinants underpinning dietary self‐management in CKD. By taking a critical‐realist pragmatic perspective of the data and mapping we identified a comprehensive set of barriers and enablers that reflect not only cognitive and informational needs, but also the emotional adjustment, social dynamics, and healthcare system influences. Our approach advances the understanding of why dietary adherence in CKD may remain challenging [8], despite widespread awareness of its clinical importance. Being pragmatic allowed us to translate our insights into intervention functions and behaviour change techniques, consistent with a pragmatic orientation to generating usable knowledge for clinical practice [26, 27].

The domains of Optimism (having a hopeful outlook that things will improve) and Intentions (a stated plan to carry out a specific action) were notably absent from the dataset, suggesting that dietary self‐management in CKD may be driven more by coping, confidence, and context than by explicit future planning or positive expectancy [17], i.e. believing that making dietary changes will help and actively deciding in advance to follow certain eating patterns for CKD in any situation. Their absence may indicate that while patients recognise the importance of diet, motivation is often reactive as it is shaped by symptoms, social pressures, and iterative trial‐and‐error, rather than proactive goal setting. This distinction is critical for intervention design, as strategies emphasising forward planning may be less impactful unless they also address the emotional and contextual realities of living with CKD.

Mapping these Theoretical Domains Framework (v2) domains [17] onto the Behaviour Change Wheel [18] emphasised the need for multi‐faceted interventions that combine Education, Enablement, and Environmental Restructuring with targeted use of Training, Persuasion, and Modelling. These functions address core barriers to dietary self‐management in CKD such as conflicting advice, low self‐efficacy, cultural misalignment of resources, and limited decision‐making support [8]. Importantly, our analysis reinforces that knowledge provision (dietary advice) alone is insufficient; interventions must also build practical skills, foster confidence, restructure healthcare environments, and mobilise social networks.

The application of the APEASE criteria to each Behaviour Change Wheel intervention function [18] provided a pragmatic lens to evaluate feasibility, acceptability, cost‐effectiveness, and equity. Education and Enablement consistently scored strongly across all criteria, reflecting their affordability, ease of delivery, and broad acceptability when culturally adapted. In contrast, Incentivisation and certain forms of Environmental Restructuring were more resource‐intensive or risked inequitable access, which implied the need for context‐sensitive adaptation. Undertaking a structured appraisal ensures that intervention design is not only theoretically robust but also deliverable within the constraints of clinical and community settings.

This study represents a theoretically distinct secondary analysis of a previously published qualitative synthesis, addressing a different research objective through the application of behavioural science frameworks. While the original review focused on identifying and co‐creating themes describing patient experiences, the present analysis extends these findings by systematically mapping them onto the Theoretical Domains Framework (v2) [17] and Behaviour Change Wheel [18] to inform intervention design. The analytical approach was specified a priori within a PROSPERO‐registered protocol, supporting transparency and methodological rigour. This structured extension enabled the translation of qualitative insights into theory‐informed, actionable targets for intervention development.

The mapping process has several limitations. First, although the Theoretical Domains Framework (v2) [17] and Behaviour Change Wheel [18] mapping followed established guidance, it inherently involves subjective judgement, and alternative mappings may be equally valid. Second, the qualitative meta‐synthesis was based on published studies, which may underrepresent certain patient groups or contexts, potentially limiting generalisability. Third, the absence of Optimism and Intentions domains may reflect the nature of the included data rather than a true lack of relevance to CKD dietary self‐management. Finally, the APEASE ratings were informed by available evidence and expert opinion but have not been empirically tested in this population, meaning the feasibility and cost‐effectiveness of recommended interventions may differ in real‐world implementation.

The author team conducting this secondary analysis substantially overlaps with that of the original qualitative synthesis [8]. This may be considered both a strength and a limitation. Familiarity with the dataset and prior immersion in the coding and theme development may have enhanced the depth, consistency, and interpretive rigour of the analysis. However, it also introduces the potential for interpretive bias, including the risk of privileging prior assumptions or being less sensitive to alternative explanations. To mitigate this, the secondary analysis followed a structured, theory‐driven approach, with iterative discussion among multiple reviewers to support reflexivity and analytical transparency.

By integrating qualitative evidence, behavioural theory, and implementation criteria, this work moves beyond describing barriers to dietary self‐management in CKD and actively specifies intervention strategies that can be transferred into tangible actions embedded into interventions. Such a process increases the likelihood that resulting dietary self‐management programmes for CKD will be both effective and sustainable, particularly if delivered in a peer‐supported, culturally competent, and digitally enabled format. Future research should test the relative impact of these intervention components in controlled settings, paying close attention to how the effects might differ between different groups of patients and stages of CKD, given the primary source of evidence for this mapping study.

## 5 Conclusion

This study provides a systematic behavioural analysis of the determinants influencing dietary self‐management among adults with CKD. By integrating the Theoretical Domains Framework (v2) with the Behaviour Change Wheel, we identified multi‐level factors: knowledge, skills, professional role, emotion, environmental context, and social influence that shape dietary adherence.

The findings extend beyond traditional education‐focused models by offering a theoretically grounded, reproducible framework for intervention design. Mapping these determinants to corresponding intervention functions and evaluating them through the APEASE criteria provides a transparent pathway for developing feasible and context‐appropriate strategies to improve dietary behaviours in CKD.

Collectively, this work advances the methodological rigour of nutrition intervention development by translating behavioural theory into actionable guidance for dietetic practice. The integrated Behaviour Change Wheel and Theoretical Domains Framework (v2) approach demonstrates potential to enhance the personalisation, scalability, and equity of dietary self‐management interventions. Future research should test the clinical effectiveness of these theoretically informed strategies in improving not only dietary behaviours but also clinical outcomes such as biochemical markers, symptom burden, and quality of life in people living with CKD.

## Author Contributions

**Andrew Morris:** conceptualisation, methodology, project administration, data curation, formal analysis, investigation, writing – original draft, writing – review and editing. **Riya Patel:** formal analysis, investigation, writing – original draft. **Deborah Lycett:** investigation, writing – review and editing.

## Funding

The authors have nothing to report.

## Conflicts of Interest

The authors declare no conflicts of interest.

## Supporting information

Supporting File

## Data Availability

Data sharing not applicable to this article as no datasets were generated or analysed during the current study.
