# Supplementary material for: Behavioural Determinants of Dietary Self‐Management in Chronic Kidney Disease: A Theory‐Informed Analysis to Inform Dietetic Interventions
Source: J Hum Nutr Diet. 2026 Jul 2;39(4):e70295. doi: 10.1111/jhn.70295 (PMC13328809; doi:10.1111/jhn.70295)
Supplement: Supplementary file 1 — Supporting File [file JHN-39-0-s001.docx]

| Version 1 | |
| --- | --- |
| Domain | Constructs |
| Knowledge | Knowledge  Knowledge about condition/scientific rationale  Schemas + mindsets + illness representations  Procedural knowledge |
| Skills | Skills  Competence/ability/skill assessment  Practice/skills development  Interpersonal skills  Coping strategies |
| Social/professional role and identity | Identity  Professional identity/boundaries/role  Group/social identity  Social/group norms  Alienation/organisational commitment |
| Beliefs about capabilities | Self-efficacy  Control—of behaviour and material and  Social environment  Perceived competence  Self-confidence/professional confidence  Empowerment  Self-esteem  Perceived behavioural control  Optimism/pessimism |
| Beliefs about consequences | Outcome expectancies  Anticipated regret  Appraisal/evaluation/review  Consequents  Attitudes  Contingencies  Reinforcement/punishment/consequences  Incentives/rewards  Beliefs  Unrealistic optimism  Salient events/sensitisation/critical incidents  Characteristics of outcome expectancies—physical, social, emotional; sanctions/rewards, proximal/distal, valued/not valued, probable/improbable, salient/not salient, perceived risk/threat |
| Motivation and goals | Intention; stability of intention/certainty of intention  Goals (autonomous, controlled)  Goal target/setting  Goal priority  Intrinsic motivation  Commitment  Distal and proximal goals  Transtheoretical model and stages of change |
| Memory, attention and decision processes | Memory  Attention  Attention control  Decision-making |
| Environmental context and resources | Resources/material resources (availability and management)  Environmental stressors  Person × environment interaction  Knowledge of task environment |
| Social influences | Social support  Social/group norms  Organisational development  Leadership  Team working  Group conformity  Organisational climate/culture  Social pressure  Power/hierarchy  Professional boundaries/roles  Management commitment  Supervision  Inter-group conflict  Champions  Social comparisons  Identity; group/social identity  Organisational commitment/alienation  Feedback  Conflict—competing demands, conflicting roles  Change management  Crew resource management  Negotiation  Social support: personal/professional/organisational, intra/interpersonal, society/community  Social/group norms: subjective, descriptive, injunctive norms  Learning and modelling |
| Emotion | Affect  Stress  Anticipated regret  Fear  Burn-out  Cognitive overload/tiredness  Threat  Positive/negative affect  Anxiety/depression |
| Behavioural regulation | Goal/target setting  Implementation intention  Action planning  Self-monitoring  Goal priority  Generating alternatives  Feedback  Moderators of intention-behaviour gap  Project management  Barriers and facilitators |
| Nature of the behaviours | Routine/automatic/habit  Breaking habit  Direct experience/past behaviour  Representation of tasks  Stages of change model |
| Version 2 | |
| Domain (definition) | Constructs |
| 1. Knowledge  (An awareness of the existence of something) | Knowledge (including knowledge of condition/scientific rationale)  Procedural knowledge  Knowledge of task environment |
| 2. Skills  (An ability or proficiency acquired through practice) | Skills  Skills development  Competence  Ability  Interpersonal skills  Practice  Skill assessment |
| 3. Social/professional role and identity  (A coherent set of behaviours and displayed personal qualities of an individual in a social or work setting) | Professional identity  Professional role  Social identity  Identity  Professional boundaries  Professional confidence  Group identity  Leadership  Organisational commitment |
| 4. Beliefs about capabilities  (Acceptance of the truth, reality or validity about an ability, talent or facility that a person can put to constructive use) | Self-confidence  Perceived competence  Self-efficacy  Perceived behavioural control  Beliefs  Self-esteem  Empowerment  Professional confidence |
| 5. Optimism  (The confidence that things will happen for the best or that desired goals will be attained) | Optimism  Pessimism  Unrealistic optimism  Identity |
| 6. Beliefs about Consequences  (Acceptance of the truth, reality, or validity about outcomes of a behaviour in a given situation) | Beliefs  Outcome expectancies  Characteristics of outcome expectancies  Anticipated regret  Consequents |
| 7. Reinforcement  (Increasing the probability of a response by arranging a dependent relationship, or contingency, between the response and a given stimulus) | Rewards (proximal/distal, valued/not valued, probable/improbable)  Incentives  Punishment  Consequents  Reinforcement  Contingencies  Sanctions |
| 8. Intentions  (A conscious decision to perform a behaviour or a resolve to act in a certain way) | Stability of intentions  Stages of change model  Transtheoretical model and stages of change |
| 9. Goals  (Mental representations of outcomes or end states that an individual wants to achieve) | Goals (distal/proximal)  Goal priority  Goal/target setting  Goals (autonomous/controlled)  Action planning  Implementation intention |
| 10. Memory, attention and decision processes  (The ability to retain information, focus selectively on aspects of the environment and choose between two or more alternatives) | Memory  Attention  Attention control  Decision making  Cognitive overload/tiredness |
| 11. Environmental context and resources  (Any circumstance of a person’s situation or environment that discourages or encourages the development of skills and abilities, independence, social competence and adaptive behaviour) | Environmental stressors  Resources/material resources  Organisational culture/climate  Salient events/critical incidents  Person × environment interaction  Barriers and facilitators |
| 12. Social influences  (Those interpersonal processes that can cause individuals to change their thoughts, feelings, or behaviours) | Social pressure  Social norms  Group conformity  Social comparisons  Group norms  Social support  Power  Intergroup conflict  Alienation  Group identity  Modelling |
| 13. Emotion  (A complex reaction pattern, involving experiential, behavioural, and physiological elements, by which the individual attempts to deal with a personally significant matter or event) | Fear  Anxiety  Affect  Stress  Depression  Positive/negative affect  Burn-out |
| 14. Behavioural regulation  (Anything aimed at managing or changing objectively observed or measured actions) | Self-monitoring  Breaking habit  Action planning |

**Table S1 The Theoretical Domains Framework**

Reproduced with no changes
